# Cardioneuroablation Restoring Atrioventricular Conduction in Brugada Syndrome: A Device-Free Approach

**DOI:** 10.1016/j.jaccas.2026.107843

**Published:** 2026-04-20

**Authors:** Fredy Chipa-Ccasani, Josep Brugada, Agustín Leonardo Luján, Estefanía Martínez-Barrios, Nuria Díez-Escuté, Erika Fernanda Merchan Pinto, Oscar Campuzano, José Cruzalegui, Andrea Greco, Georgia Sarquella-Brugada

**Affiliations:** a Arrítmies Pediàtriques, Cardiologia Genètica I Mort Sobtada, Malalties Cardiovasculars en el Desenvolupament, Institut de Recerca Sant Joan de Déu, Esplugues de Llobregat, Barcelona, Spain; b Pediatric Arrhythmias, Inherited Cardiac Diseases and Sudden Death Unit, Hospital Sant Joan de Déu, Barcelona, Spain; c School of Medicine, University of Girona, Girona, Spain; d Pediatrics Department, School of Medicine, University of Barcelona, Barcelona, Spain

**Keywords:** atrioventricular block, Brugada syndrome, cardioneuroablation

## Abstract

**Background:**

Brugada syndrome (BrS), traditionally associated with ventricular arrhythmic risk, may also involve conduction disturbances. *SCN5A* deleterious variants predispose to bradyarrhythmias and atrioventricular block, posing the challenge of pacemaker implantation in young patients.

**Case Summary:**

A 17-year-old girl with BrS and *SCN5A* p.(Glu901Lys) pathogenic variant presented with recurrent presyncope, dyspnea, and more than 3,000 second-degree atrioventricular blocks documented on 24-hour Holter monitoring. Atropine administration reversed the block, confirming a functional origin. Biatrial approach cardioneuroablation (CNA) guided by electroanatomical mapping without fluoroscopy restored normal conduction and improved sinus rate. After 12 months, the patient remained asymptomatic and free of bradyarrhythmias.

**Discussion:**

CNA restores autonomic balance and corrects functional bradyarrhythmias in BrS, avoiding permanent pacing. Success of CAN depends on accurate identification of the functional mechanism and expertise in autonomic mapping.

**Take-Home Messages:**

In BrS, vagally mediated functional bradyarrhythmias may respond to atropine testing. CNA offers a physiological and durable alternative to pacemaker implantation in young patients with functional atrioventricular block.

## Graphical Abstract

**Figure:**
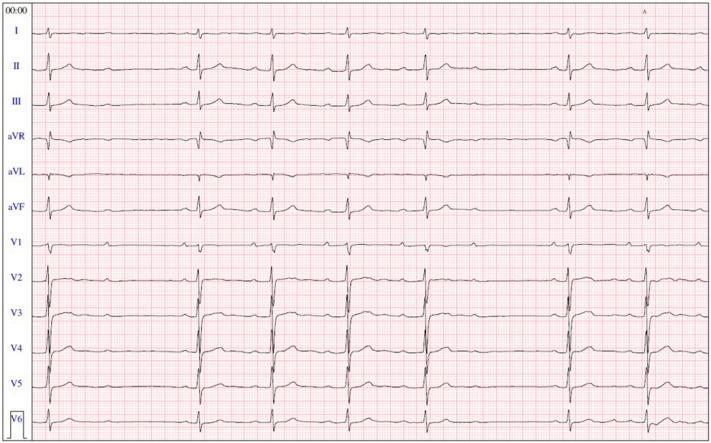

## History of Presentation

A 17-year-old girl who had been previously diagnosed with Brugada syndrome (BrS) was referred for recurrent episodes of lightheadedness and progressive dyspnea. Five years earlier, the patient had a positive ajmaline test and a negative electrophysiological study for ventricular tachycardia induction, with no baseline conduction abnormalities.

**Take-Home Message**

• This case underscores the value of CNA as a physiological alternative in adolescents with BrS and functional AV block, avoiding pacemaker implantation and achieving long-term normalization of AV conduction.

**Figure 1:**
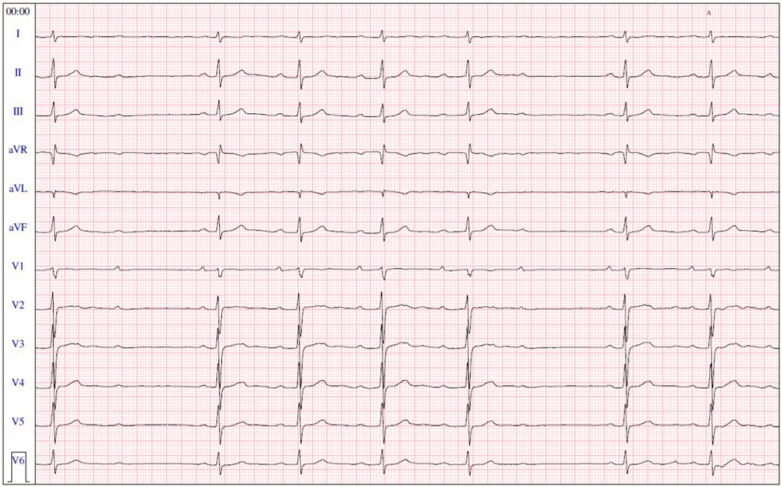
Baseline Electrocardiogram Baseline electrocardiogram showing sinus rhythm and type I atrioventricular second-degree block. No type I Brugada pattern is observed in the right precordial leads.

At our center, the baseline electrocardiogram showed sinus rhythm with spontaneous Wenckebach phenomenon, without an evident Brugada pattern (Figure 1). A 24-hour Holter monitor recorded more than 3,000 episodes of second-degree atrioventricular (AV) block, leading to referral for pacemaker evaluation. The patient reported significant limitation in daily and athletic activities, with considerable emotional distress due to fatigue and recurrent symptoms.

The patient carried the genetic variant c.2701G>A; p.(Glu901Lys) in the *SCN5A* gene (transcript NM_000335.5), previously identified within her family. This rare variant was classified as pathogenic following current American College of Medical Genetics and Genomics/Association for Molecular Pathology guidelines.^1^, ^2^, ^3^

## Family History

The index case in the family was the older sister, who was diagnosed with BrS after a spontaneous type I Brugada electrocardiogram pattern was detected during a routine evaluation for a cardiac murmur. A wide-ranging genetic panel was performed including all genes currently associated with BrS as well as inherited arrhythmogenic syndromes. Only the rare variant c.2701G>A; p.(Glu901Lys) was identified in *SCN5A*.

Family history identified clinically asymptomatic parents, and genetic analysis in both showed that the variant was inherited from her mother. The maternal grandmother carried the same variant despite no clinical symptoms reported. In addition, a maternal uncle experienced sudden cardiac death at 33 years of age while dining, suggesting a possible hereditary arrhythmogenic substrate.

## Differential Diagnosis

Progressive intrinsic AV block, functional AV block mediated by increased vagal tone, and sinus node dysfunction associated with BrS were included in the differential diagnosis.

## Investigations

**Figure 2:**
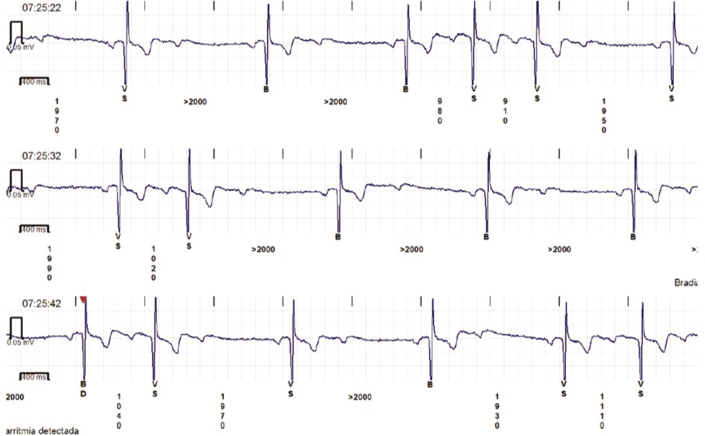
Implantable Loop Recorder Tracing Implantable loop recorder tracing obtained before cardioneuroablation showing episodes of type I and type II atrioventricular block with pauses exceeding 2,000 ms.

An implantable loop recorder (ILR) revealed multiple episodes of AV block with pauses exceeding 2,000 ms (Figure 2). The exercise stress test demonstrated partial reversal of second-degree AV block. The electrophysiological study showed a severe supra-Hisian conduction system disturbance, with complete reversal of the block after atropine administration, confirming its functional origin.^4^

## Management

**Figure 3:**
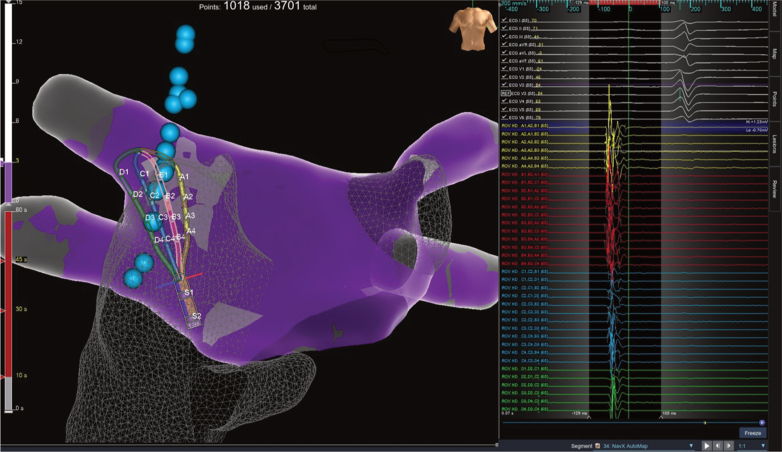
Fractionated Electrograms and Electroanatomical Mapping On the left, fractionated electrograms recorded with omnipolar signal from the HD Grid catheter at the superior paraseptal ganglionated plexus. Electroanatomical map on the right shows in gray the areas with fractionated electrograms used as reference for ablation; light blue dots indicate the course of the phrenic nerve.

**Figure 4:**
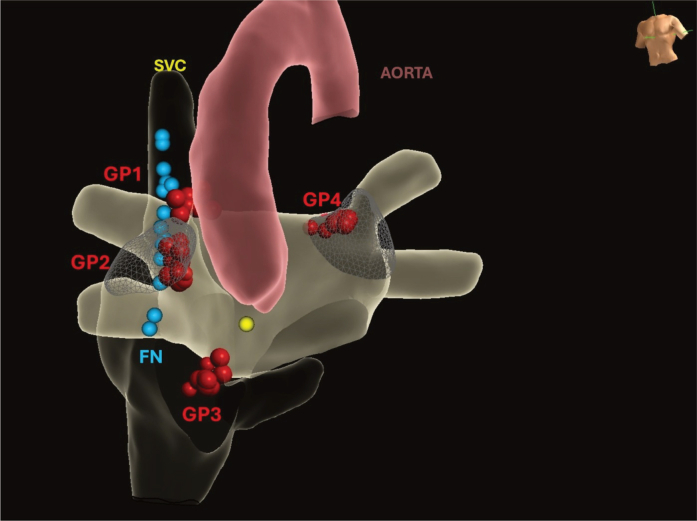
3-Dimensional Reconstruction of the Right and Left Atria Red dots mark ablation sites, light blue dots delineate the course of the phrenic nerve (FN), and the yellow dot indicates the location of His bundle. The superior vena cava (SVC), aorta, and ganglionated plexi (GP1–GP4) are also identified.

Pacemaker implantation was deferred for symptomatic AV block, and cardioneuroablation (CNA) was performed under general anesthesia. The procedure included transseptal puncture guided by transesophageal echocardiography, high-density biatrial mapping using an HD Grid catheter and EnSite X navigation system (Abbott Cardiovascular), and detailed aortic anatomical mapping to optimize localization and delineation of the ganglionated plexi (Figure 3). Areas with fractionated electrograms consistent with autonomic activity were identified (Video 1). Ablation was performed using an irrigated TactiFlex catheter (30 W, ≤20 seconds per application, impedance drop 10%-15%) targeting the aorto–superior vena cava (GP1), superior (GP2) and inferior (GP3) paraseptal, and left superior (GP4) ganglionated plexi, as shown in Figure 4, following previously described CNA strategies.^4^, ^5^, ^6^, ^7^

During radiofrequency delivery, the heart rate increased from 50 to 85 beats/min. Immediately after CNA, the AH interval decreased from 145 ms to 74 ms, the HV interval remained stable (57-56 ms), and the Wenckebach point shortened from 1,000 ms to 460 ms. Subsequent administration of 2 mg of atropine produced no changes in heart rate or AH and HV intervals, confirming complete parasympathetic denervation.^4^, ^5^, ^6^, ^7^ No complications occurred during the procedure.

## Follow-Up

The patient was discharged the same day without complications and was prescribed aspirin 100 mg/day for 1 month. At 4 weeks post-procedure, she showed marked clinical improvement, with resolution of dyspnea and fatigue. A significant emotional recovery was also noted, with reduced anxiety and restored confidence to resume normal activities, resulting in an overall improvement in quality of life.

**Figure 5:**
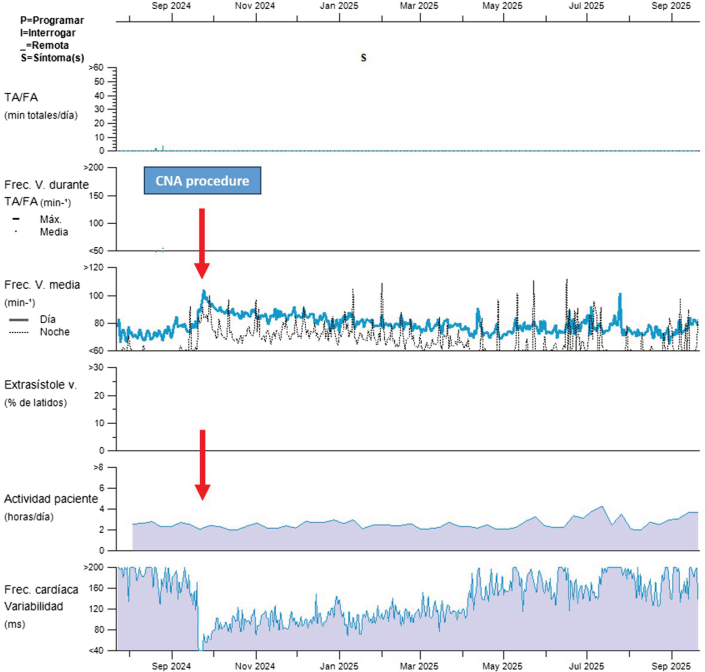
Longitudinal Recording From ILR Longitudinal recording from implantable loop recorder (ILR) showing immediate and sustained increase in mean heart rate after cardioneuroblation, with a progressive rise in physical activity. An initial decrease in heart rate variability, consistent with effective vagal denervation, is followed by partial recovery without reaching preprocedural values.

Continuous follow-up using an ILR revealed an immediate and sustained increase in mean heart rate after CNA, along with a progressive increase in daily physical activity (Figure 5). Heart rate variability showed a sudden postprocedural decline—consistent with effective vagal denervation—followed by gradual recovery that did not reach baseline levels. This pattern reflects partial and stable autonomic modulation rather than complete denervation, in line with previously reported post-CNA vagal readaptation.

**Figure 6:**
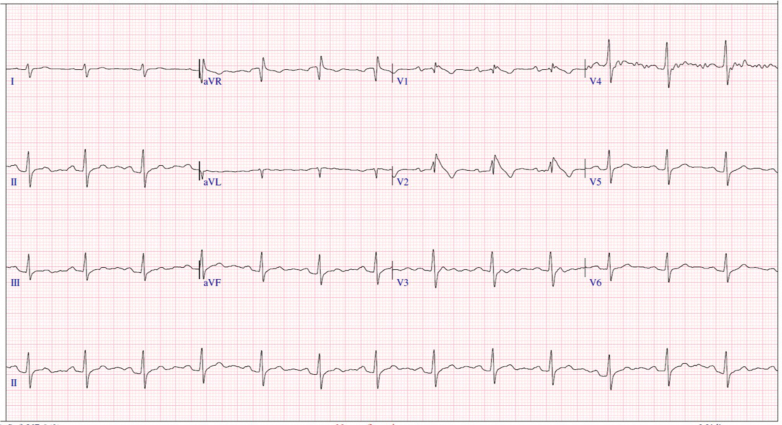
6-Month Post-CNA Electrocardiogram Electrocardiogram obtained 6 months post-cardioneuroblation (CNA) showing stable sinus rhythm with a type I Brugada pattern, without pauses or atrioventricular conduction abnormalities.

At 6 months, electrocardiogram showed stable sinus rhythm with a spontaneous type I Brugada pattern and no conduction abnormalities (Figure 6). At 12 months, the patient remained asymptomatic, with no syncope, pauses, or arrhythmias, confirming the clinical and autonomic success of CNA.

## Discussion

CNA, first described by Pachón et al in 2005,^4^ has emerged as an effective therapeutic alternative to pacemaker implantation in patients with functional bradyarrhythmias. Subsequent studies confirmed the ability of CNA to modulate autonomic balance and restore AV conduction in cases of functional block.^5^, ^6^, ^7^, ^8^ In BrS, predominant vagal tone can exacerbate both bradyarrhythmias and ventricular arrhythmic risk, and autonomic influences may modulate the underlying arrhythmogenic substrate.^4^, ^5^, ^6^, ^7^, ^8^ Recent reviews have outlined the expanding role of CNA in neurally mediated syncope, sinus dysfunction, and AV block.^8^, ^9^, ^10^ This case highlights the importance of distinguishing arrhythmic syncope from cardioinhibitory reflex in young patients carrying the *SCN5A* pathogenic variant, thus avoiding unnecessary permanent device implantation.

The p.Glu901Lys (rs199473174; minor allele frequency: 0.0001%) substitution alters a highly conserved residue within the pore domain of the cardiac sodium channel, in close proximity to the selectivity filter, a region essential for maintaining proper ion permeability and channel gating. This variant has been reported in association with BrS in several cases, and functional studies demonstrated that it causes a loss of function, consistent with the pathophysiological mechanism of BrS.^1^, ^2^, ^3^

BrS, classically linked to ventricular tachyarrhythmias, may also involve AV conduction disturbances. Deleterious *SCN5A* variants reduce sodium current in the AV node and His-Purkinje system, predisposing to PR interval prolongation or intermittent AV block.^1^, ^2^, ^3^ Some AV block cases in this context are functional, mediated by excessive vagal activity, and can be reversed with pharmacologic testing or CNA.^4^, ^5^, ^6^, ^7^, ^8^

CNA restores autonomic balance by reducing parasympathetic influence on the sinus and AV nodes, thereby normalizing chronotropic and dromotropic responses.^4^, ^5^, ^6^, ^7^, ^8^ In young patients, this approach is particularly appealing because it avoids permanent pacemaker implantation and its long-term complications. In our patient, follow-up with an ILR demonstrated an immediate and sustained increase in heart rate after CNA, with no recurrence of bradyarrhythmias or pauses, reflecting effective and durable parasympathetic denervation. These findings align with previous studies showing stable restoration of autonomic tone and AV conduction after CNA.^5^, ^6^, ^7^, ^8^

## Conclusions

CNA is an effective and safe therapeutic alternative for adolescents with BrS and functional AV block, restoring autonomic balance and normalizing conduction without the need for permanent devices. The correct identification of a functional origin, through atropine response and autonomic assessment, is essential for appropriate patient selection. Controlled vagal modulation may translate into clinical stability, improved quality of life, and prevention of unnecessary pacemaker implantation in young patients with channelopathies.^4^, ^5^, ^6^, ^7^, ^8^

## Funding Support and Author Disclosures

The authors have reported that they have no relationships relevant to the contents of this paper to disclose.

## Appendix

Video 1 Three-Dimensional Electroanatomical Mapping of Right Atrium and Superior Vena Cava Shows Radiofrequency Delivery at the Aorto–Superior Vena Cava Ganglionated Plexus Simultaneous surface electrocardiogram and intracardiac electrograms demonstrate progressive RR interval shortening and sinus rate acceleration during ablation, consistent with an immediate vagal withdrawal and effective autonomic modulation.
